# Live Cells as Dynamic Laboratories: Time Lapse Raman Spectral Microscopy of Nanoparticles with Both IgE Targeting and pH-Sensing Functions

**DOI:** 10.1155/2012/390182

**Published:** 2012-06-24

**Authors:** Kristy L. Nowak-Lovato, Kirk D. Rector

**Affiliations:** ^1^Decision Applications Division, Los Alamos National Laboratory, Los Alamos, NM 87545, USA; ^2^Chemistry Division, Los Alamos National Laboratory, Los Alamos, NM 87545, USA

## Abstract

This review captures the use of live cells as dynamic microlaboratories through implementation of labeled nanoparticles (nanosensors) that have both sensing and targeting functions. The addition of 2,4-*ε*-dinitrophenol-L-lysine (DNP) as a Fc*ε*RI targeting ligand and 4-mercaptopyridine (4-MPy) as a pH-sensing ligand enables spatial and temporal monitoring of Fc*ε*RI receptors and their pH environment within the endocytic pathway. To ensure reliability, the sensor is calibrated *in vivo* using the ionophore nigericin and standard buffer solutions to equilibrate the external [H^+^] concentration with that of the cell compartments. This review highlights the nanosensors, ability to traffic and respond to pH of receptor-bound nanosensors (1) at physiological temperature (37°C) versus room temperature (25°C), (2) after pharmacological treatment with bafilomycin, an H^+^ ATPase pump inhibitor, or amiloride, an inhibitor of Na^+^/H^+^ exchange, and (3) in response to both temperature and pharmacological treatment. Whole-cell, time lapse images are demonstrated to show the ability to transform live cells into dynamic laboratories to monitor temporal and spatial endosomal pH. The versatility of these probes shows promise for future applications relevant to intracellular trafficking and intelligent drug design.

## 1. Introduction

The chemical concentrations, within the lumens of cellular organelles, such as endosomes, lysosomes, mitochondria, the Golgi apparatus, and the endoplasmic reticulum, are frequently different from the cytoplasm. The locations and chemical differences in these cellular compartments are now being individually interrogated with an ever-expanding suite of techniques. The vast majority of these reagents should be considered fluorescence labels as their spectral responses are integrated to provide contrast in the confocal image [1, 2]. Frequently, however, it is desirable to have a sensor, rather than a label, in order to measure metabolite concentrations, including Ca^2+^, NO, Na^+^, and pH [3]. Generally, the sensor must be employed in the spectral domain as features in the spectrum change in response to the changing analyte concentration, chemical or oxidative state. 

It is also possible to perform experiments using Raman scattering as the contrast mechanism in cellular imaging. Raman spectroscopy or imaging provides chemically specific information based upon unique vibrational signatures of the species present. Therefore, recording of the spectra of a cell can give detailed information of the chemical species present. Unfortunately, the Raman effect is weak, with molecular cross-sections in the 10^−29^ to 10^−32^ cm^2^ range, significantly lower than those of good fluorescence labels which are in the 10^−16^ cm^2^ range [4]. An inherent benefit of Raman microscopy over fluorescence is that it records the natural abundance of chemicals present and, therefore, is a label-free technique. However, if one was willing to forego this advantage, it becomes possible to use Surface-enhanced Raman scattering (SERS)-based nanoparticles as labels or sensing agents [5–13]. SERS provides an increase in the Raman scattering signal by many orders of magnitude [9, 14–16]. This enhancement makes it possible to access the chemical specificity inherent in Raman spectroscopy with low detection limits and/or fast acquisition speeds. In addition, it enables nearly limitless flexibility to design experiments based upon the ligands chosen for the nanoparticle surface. In general, multiple ligands are possible, including ligands that serve to cause the cell to deliver nanoparticles to specific locations or systems and ligands which record the concentrations of specific metabolites when present. The creation of a targeted nanosensor, one which has both functions, creates a novel tool to turn cells into dynamic laboratories. Further, the most significant advantage may ultimately be the ability to record time-lapse spectral microscopy of whole live cells as they respond to stimuli for substantially longer periods of time over fluorescence microscopy that tend to suffer from photobleaching.

Nanosensors are created based upon choices of a colloidal particle platform, a targeting ligand, and a reporter molecule. The nanoparticle platforms generally include noble metals, particularly of Ag [17], and Au [18, 19], as well as a variety of geometries including hollow shell [20] and core/shell [21, 22] particles. For targeting ligands, generally researchers have used localization peptides [23] or antibodies (IgE) to deliver the nanoparticles to specific locations or subcellular systems [24–26]. For the reporter ligands, the most demonstrated analyte concentration studied with SERS nanosensors is pH. Roughly speaking, the sensitivity of the nanosensor will be within a couple orders of magnitude of the dissociation constant,  *k*_*D*_, of the reporter molecule used. The ligands chosen are based upon the ability to coat the metal surface and have unique Raman vibrational signatures as well as sensitivity and specificity to the analyte of interest. For pH, various researchers have demonstrated basic [27], neutral [17, 20], and acidic-range [10–12, 28, 29] sensors based on different ligands. In particular, for measurements of acidic concentrations (3 < pH < 7), it has been shown that use of 4-mercaptopyridine (4-MPy) is an effective strategy [7, 21]. The focus of this work is on the cell's dynamic regulation of acidic pH within the endosome-lysosome system, which is necessary to support degradative enzymes as well as the proper sorting of receptors and ligands following endocytosis [30–32]. Endosomal sequestration of weakly basic compounds such as daunorubicin, possibly through a pH-partitioning mechanism, may be a factor in drug resistance [33]. Low pH within the endosomal-lysosomal system can also play a role in development of disease states [34–37]. 

To be able to employ live cells as dynamic microlaboratories, it is important that entire hyperspectral maps be recorded. However, point spectral Raman mapping is generally too slow to be relevant for most biological systems. One approach to this goal is the use of a so-called “push-broom” Raman microscope [38], where the excitation light is cylindrically, rather than spherically, focused to a line image across the sample, and the resulting Raman line image is then dispersed onto a CCD camera. The cylindrically focused line image allows for the collection of hundreds of spectra, along a single spatial line, to be recorded simultaneously. The sample is then stepped across the line to build up the other spatial axis, resulting in an entire hyperspectral 3D map in a couple of minutes, depending on signal strength. The spectral maps consist of two spatial dimensions (*x*, *y*), defining the area of interest, and the spectral dimension (*λ*) used to identify the chemical composition of the material at each pixel of the image. The map is then reacquired sequentially to build up dynamic spectral movies (*x*, *y*, *λ*, *t*). These time lapse spectral movies allow for the identification of cells in* statis* as well as the recording of them responding to external stimuli. The ability to monitor changes in intraluminal pH, in the context of cargo moving through the endosomal-lysosomal system or in response to causal on/off external stimuli, could lead to novel strategies toward drug development and understanding of pathogen survival and other disease states [39–43].

Recently, it has been shown that a targeted SERS nanosensor with both IgE-targeting and pH-sensing functions is capable of measuring the pH of intracellular compartments harboring internalized IgE receptors, using the RBL-2H3 mast cell line as a model system. This sensor is based upon a Au@Ag core shell particle with MPy as a reporter molecule and 2,4 *ε*-dinitrophenol-L-lysine (DNP) as a targeting molecule for IgE bound to the high affinity IgE receptor, Fc*ε*RI [10, 12]. Subsequently, the application of this nanosensor to measure the pH of endosomal compartments as sensor-bound receptors traffics through the endosome-lysosome system while the cell exposed to dynamic external stimuli has been reported [11]. It was demonstrated that the nanosensors are resilient to changes in temperature, by lowering the temperature of cultured cells from the physiologically relevant temperature of 37°C to 25°C; this procedure also slows the transit of endocytosed receptors. More importantly, it was also demonstrated that the targeted nanosensors report changes in luminal pH after treatment of cells with the H^+^ flux inhibiting drugs, amiloride and bafilomycin. Importantly, the Fc*ε*RI-targeted pH SERS nanosensors enable the direct localization of vesicles bearing Fc*ε*RI receptors and also report the dynamic changes in endosomal vesicle pH in response to these drugs. The compartments recover their normal pH after the removal of these drugs from the media on the timescale of 10s of minutes. This technology has the potential to create new techniques useful in the fields of medicine, pharmacology, and biotechnology. 

## 2. Experimental Section

### 2.1. Chemicals and Materials

Au colloids at 60 nm diameter were purchased from Ted Pella, *Inc*. (p/n 15708-6, Redding, CA) at 2.6 × 10^10^ particles per mL. The Au colloids were enhanced with a Ag coating by the addition of LI silver enhancing and initiator agents from Nanoprobes, Inc. (p/n 2013, Yaphank, NY, USA). Silver enhancement was performed at a 1 : 1 ratio of both initiator and enhancer. The enhancement solution was added to the colloids for two minutes, centrifuged at 13,000 rpm for five minutes, and then washed with deionized H_2_O. MPy (p/n 148202, Sigma Aldrich Chemicals, St. Louis, MO) and DNP (MP Biomedical, Solon, OH, USA) used without further purification and aqueous solutions were prepared at 1 mM. BSA-DNP and IgE antibody was acquired via a generous gift from the Oliver-Wilson laboratory at the University of New Mexico. Nigericin, KCl, MgCl_2_, CaCl_2_, and glucose were purchased from Sigma Aldrich chemicals (St. Louis, MO, USA) and used without further purification. Bafilomycin (Sigma) was dissolved in EtOH and used at a concentration 200 nM [44]. Amiloride (MP Biomedical, *Inc.*) was dissolved in dH_2_O and used at a concentration of 1 mM [45].

### 2.2. Cell Culture

 For cellular experiments, RBL-2H3 cells were cultured in MEM media supplemented with 10% fetal clone III (p/n SH3010902, Thermo Scientific Hyclone, Logan, UT), 90 I.U./mL of penicillin (ATCC, Manassas, VA, USA) and 90 *μ*g/mL streptomycin (ATCC, Manassas, VA, USA), with an additional 2 *μ*M of L-glutamine (Sigma Aldrich, St. Louis, MO, USA). RBL-2H3 cells are an adherent cell line that was grown in a 5% supplied CO_2_ incubator at 37°C to 80% confluency and then passaged. Cells were primed with anti-DNP-specific IgE (1 *μ*g/mL) 18–24 hrs and kept at 37°C and 5% CO_2_ prior to use. Imaging experiments with the targeted nanosensors employ sonication of colloids for five minutes and filtering through a 0.2 *μ*m filter and added to the cells at 10–100x particles per cell number. After five minutes, the cells are then washed 2x with phosphate-buffered solution, to eliminate most of the residual nanoparticles left outside of the cell, and then freshly warmed Hanks buffer is applied. The buffer consists of Hanks, HEPES, BSA, glucose, MgSO_4_, and NaHCO_3_. The cover slips with adherent cells are then added to the environmental chamber vessel (Carl Zeiss, Inc., Thornwood, NY, USA) equilibrated at 25°C or 37°C and 5% CO_2_ for imaging. 

### 2.3. Degranulation Assay

To demonstrate biological activity of the IgE receptor-mediated endocytosis nanosensor, a degranulation assay was performed. In this assay, *β*-hexosaminidase was used to hydrolytically cleave substrate p-nitrophenyl-N-acetyl-*β*-D-glucosaminide (N9376, Sigma) and cause a color change that can be detected. The assay was performed in 24 well culture plates. Positive control for the assay is done by lysing whole cells with 1% Triton. 

### 2.4. Microscope Layout and Imaging

The microscope-imaging system is based upon a Carl Zeiss Axiovert 135TV inverted microscope with an Epiplan 10x (N.A. 0.20), a LD Plan-achroplan 40x (N.A. 0.60 and a C-apochromat 63x (N.A. 1.2) water immersion objective. It was this last objective primarily used for the SERS experiments. A xenon arc lamp (XBO 75, Carl Zeiss, Thornwood, NY, USA) was used to illuminate the sample for bright field visualization in transmission mode, by video camera, using transfer optics in the trinocular head. (Optem 70XL, Labtek, Campbell, CA) The total zoom factor of the transfer optics was 0.5x in addition to the objective used. An Infinity 2-2 monochrome (Lumenera Corp., Ottawa, ON, USA) camera was used to acquire the visible image using an acquisition rate of approximately 200 ms/frame and no detector gain. The visible image resolution was 1616 × 1216 pixels. The camera was connected to the personal computer through a USB 2.0 connection, and the image was captured using the Infinity Capture software and saved as a  .tiff file format. Raman experiments were performed using 514.5 nm light from a Spectra Physics 177-G01 air-cooled argon ion laser. The excitation light was separated from other Ar^+^ laser lines using a 300 ln/mm dispersive grating and a ~1 mm spatial filter several feet away. The laser light is expanded and collimated using a 1 : 2 Keplerian telescope. It is coupled into the back of the microscope objective with an antireflection-coated plano-convex BK-7 150 mm focal length cylindrical lens (CKX150AR.14, Newport Corp. Irvine, CA, USA), a Raman edge dichroic (z514rdc no. 105366, Chroma Technology, Rockingham, VT, USA) and a 50.8 mm diameter, 150 mm focal length biconvex spherical anti-reflection coated doublet tube lens (PAC086AR.14, Newport Corporation, Irvine, CA). The laser light is focused to a line which is approximately 0.5 *μ*m wide and 50 *μ*m tall and a total power at the sample of 10 mW for the experiments detailed in this paper. The Raman signal is captured with the same objective and tube lens and is passed through the edge dichroic. The signal is transferred using two additional 150 mm spherical lenses and reimaged onto the 100 *μ*m-wide slit of a 0.25 m f/2.2 imaging spectrograph (Holospec 2.2, Kaiser Optical Systems, Inc, Ann Arbor, MI, USA). The signal is dispersed by a volume holographic grating (HSG-514.4-LF, Kaiser) and imaged with a LN_2_-cooled CCD array detector (LN/CCD-1024E, Princeton Instruments, Trenton, NJ, USA). Each image of the CCD records spectral and “*Y*” spatial information of the sample. The spectral calibration of the CCD was performed using a Ne standard lamp (Neon 6032, Newport, Irvine, CA, USA) and confirmed with an Hg standard lamp (Newport). The spectral resolution of the spectrograph is approximately 2 cm^−1^. The spatial axis was calibrated using a resolution test target, USAF-1951 using lines in the 6th group. The spatial axis was corrected for spherical aberrations using the Hg standard lamp and custom-written Labview code (National Instruments, Austin, TX, USA). In addition, the creation of a flat field correction was performed to remove the roughly Gaussian-shaped power dependence along the laser line focus using a NIST Relative Intensity Correction Standard (NIST 2243). Individual spectra were acquired by integration along the vertical axis, and background spectra were acquired under the same conditions using a microscope coverslip only. The data were acquired using custom Labview code and stored in ASCII format on a personal computer. In order to acquire spectral image cubes, the frequency and “*Y*” spatial axis were acquired and saved, and the sample was moved across the excitation source by stepping the computer controlled microscope stage (MS-2000, Applied Scientific Instrumentation, Eugene, OR, USA), to sequentially build up the “*X*” spatial access of the cube. The synchronization of the CCD camera and the stage were performed using the same Labview code. All cellular experiments were performed with an independently controlled environmental chamber (Carl Zeiss, Inc., Thornwood, NY, USA) mounted to the stage.

### 2.5. Electron Microscopy

Scanning electron micrographs were acquired using a FEI Quanta 200FEG operating at an accelerating voltage of 30 kV. All imaging was performed with a chamber pressure of ~100 Pa of water vapor to avoid charging and to reduce sample dehydration. Images were taken to examine the size and morphology of the nanoparticles during development. Samples were mounted on glass slides. For transmission electron microscopy (TEM), cells were fixed with 2% glutaraldehyde in cacodylate buffer (pH 7.4), postfixed with osmium tetroxide, dehydrated in ascending alcohols, and embedded in Epon. Due to the size of the Au@Ag nanosensors, samples were sectioned with a 100–300 nm thickness on a Leica Ultracut UCT. Samples were stained with uranyl acetate and lead citrate and observed on a Hitachi 7500 electron microscope.

### 2.6. Surface-Enhanced Raman Scattering pH Calibration

*In vivo* calibration experiments employ the addition of 10 *μ*m nigericin, 140 mM KCl, 1 mM MgCl_2_, 2 mM CaCl_2_, 5 mM glucose, and 20 mM phosphate-citrate buffers of varying pH (4–8). Once the calibration solution was added, cells were returned to the incubator for ten minutes. After pH equilibration, targeted nanosensors were added to the cells, and whole images were taken of all particles found to gain entry inside the cells after ten minutes of addition. Cellular images representing approximately 100 0.5 *μ*m × 0.5 *μ*m-sized pixels with nanosensors were used. These images were processed to obtain individual spectra as indicated in the data analysis section. Once individual data points were collected for each pH value, the data was fit to a sigmoidal curve. 

### 2.7. Data Analysis Procedures

 The data analysis procedures were performed using custom written Labview code. All pixels of the hyperspectral image cube were subject to the same correction procedures including wavelength calibration, flat field correction, background subtraction, CCD bias voltage subtraction, and spherical aberration removal. The images were thresholded by eliminating any spectral pixel information with a signal to noise ratio of less than 2.5. The signal-to-noise ratio was calculated by taking the highest intensity count from the integrated peak at 1572 cm^−1^ to 1599 cm^−1^ subtracted from the baseline value at that point, divided by the highest intensity count from the noise at 1800 cm^−1^ to 1827 cm^−1^ subtracted from the baseline value at that point. Any pixel with a signal-to-noise ratio below 2.5 was considered to not have any useful information and was removed from the image. The two spectral peaks of interest were then integrated from 1572 cm^−1^ to 1599 cm^−1^ for the nonprotonated peak and 1599 cm^−1^ to 1626 cm^−1^ for the protonated peak. Because the Raman spectra often occur on a weak fluorescence background, the integrated limits were evaluated against a linearly sloping background defined as the spectral intensity at 1000 cm^−1^ and 2000 cm^−1^. Because the peaks of interest are so close spectrally, variably sloping baselines that may arise from the cellular autofluorescence can be effectively removed without changing the intrinsic ratio of the peaks. 

## 3. Results and Discussion

**Figure 1:**
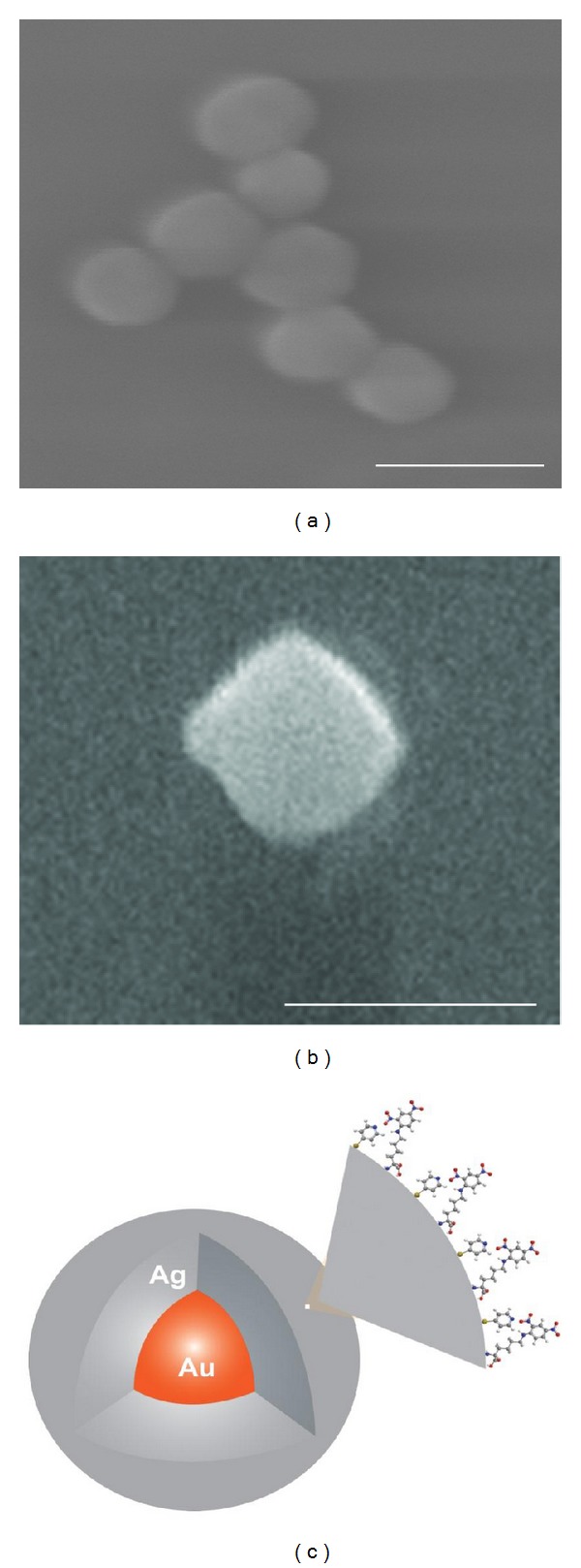
(a) Scanning electron microscope images of a few of the commercial Au colloidal particles. Scale bar is 60 *μ*m. (b) Composite SEM image of “developed” gold-silver core-shell colloidal particles. Ag layer varies from a few nm to a few 10s of nm in thickness, resulting in sizes varying from 70 to 150 nm. Scale bar is 100 *μ*m. (c) Schematic of the targeted nanosensor. Au@Ag nanoparticle with DNP and 4-MPy ligands, not drawn to scale. (a and b) Reprinted with permission from Nowak-Lovato et al. [11] (copyright 2009 Society for Applied Spectroscopy).

Scanning electron micrographs of the nanoparticles are shown in Figure 1 before (a) and after (b) development with silver. These core-shell particles are denoted Au@Ag based on similar geometries reported elsewhere [21]. After development, the particles ranged in size from approximately 70 nm to 150 nm. Any possible larger aggregates were removed via filtration using a 200 nm syringe filter. The silver development strategy proved to be a rapid, cost-effective method for generating large quantities of silver-based particles in the 100 nm range.  Figure 1(c) is a schematic illustration of the nanosensor design. The drawing is not to scale, but demonstrates the Au core with Ag surface coating as well as the addition of multiple 4-MPy ligands and multiple DNP ligands on the Ag surface. It may be of interest to put an outside boundary on the numbers of ligands on the surface of the nanoparticle. The widths of  4-MPy (between the meta hydrogens) is 0.42 nm, while the DNP width (between the nitro moiety and the H para to that) is 0.60 nm. Assuming a uniform spherical nanoparticle with a diameter of 100 nm yields an effective surface area of 31,415 nm^2^. Given equal numbers of both ligands on the surface', and that they are rigid, space filling, the maximum numbers of ligands is approximately 30,800 ligand molecules per nanoparticle. As there are many assumptions here in this idealized case, it is most likely that this calculation represents the maximum case, and the actual number is probably less than that. For the creation of targeted nanosensors with both a targeted and sensing capability, it is important to determine the degree with which either of the ligands interferes with the other's functionality.

### 3.1. Testing the Nanoparticle Targeting Ligand

To first test the effect of the presence of 4-MPy on the ability of a DNP particle to be targeted to the endosomal pathway of cell lines, a fluorescence-based degranulation assay was performed. Cellular degranulation upon stimulation of the IgE Fc*ε*RI-mediated pathway will result in the cell's secretion of constituents including the granular enzyme *β*-hexosaminidase [46]. Release of *β*-hexosaminidase is used in this assay to hydrolytically cleave an externally added substrate molecule, p-nitrophenyl-N-acetyl-(3-D-glucosamine), and cause a detectable color change. The assay was used to compare Au@Ag-DNP particles with previously measured bovine serum albumin (BSA)-DNP particles. 

**Figure 2:**
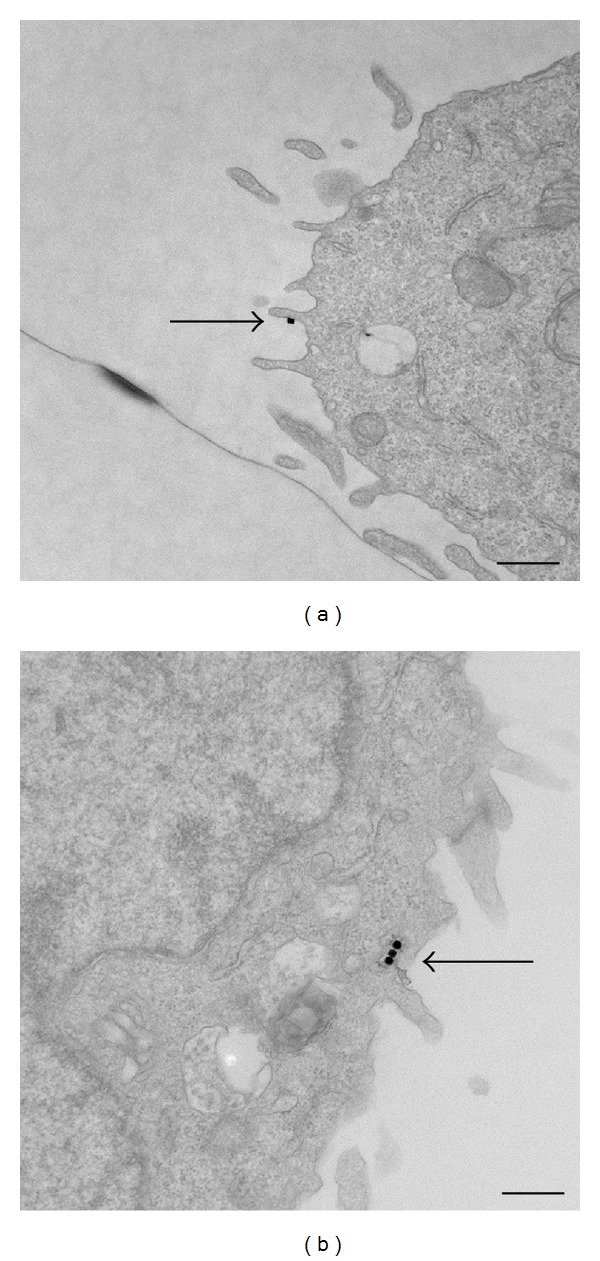
Transmission electron microscopy images of RBL-2H3 cells (a) 5 min after incubation of nanosensors and (b) 60 min after incubation with nanosensors. *Arrows* are added to pinpoint locations of nanosensors within the cells. *Bars* = 500 nm. Reprinted with permission from Nowak-Lovato et al. [11] (copyright 2010 Springer).

Assay data has been reported previously [10]. Spontaneous release resulting from IgE-sensitized cells, without the addition of BSA-DNP, shows a response at 5%, which is used as the negative control. A 5% release comparable to spontaneous release was demonstrated with the use of Au@Ag-4MPy nanoparticles, lacking the targeting DNP component. Decreasing concentrations of BSA-DNP show a corresponding drop in percentage release which serves as a positive control and generally agree to literature values for this cell line [47, 48]. Particles of Au@Ag-DNP show a 55% granule release from whole cells, which is comparable to that seen with 10 *μ*g of BSA-based DNP particles. When DNP: 4-MPy mixtures of 1 : 1, 3 : 1, and 1 : 3 were used to coat the Au@Ag nanoparticle surface, degranulation percentages were seen at 30%, 37%, and 27%, respectively, which is comparable to 10 ng of BSA-DNP. This drop in efficiency can be attributed to the decrease in concentration of the DNP on the particle surface because of the presence of 4-MPy. The 1 : 1 ratio (DNP/4-MPy) nanoparticles were chosen for these experiments, as a reasonable balance between the ability of DNP to target the nanosensor to the endosomal pathway, with high enough SERS signal levels from 4-MPy, to enable reasonably fast image acquisition.  Figure 2 demonstrates internalization of nanosensors using TEM images.  Figure 2(a) shows a high-resolution image of a RBL-2H3 cell after 5 min exposure to the nanosensors. The arrow points to the location of an individual nanosensor, seen as a black (electron-dense) dot bound to the surface of the cell, due to specific binding of DNP portion of the nanosensor to at least one IgE-bound receptor.  Figure 2(b) illustrates a similar image of a cell exposed to a five-minute pulse of nanosensors, followed by a sixty-minute chase to allow for internalization of sensor-bound receptors prior to fixation and processing for TEM. In this photo, the arrow points to three internalized nanosensors within an endocytic compartment just under the membrane surface.

### 3.2. Testing the Nanoparticle Sensing Reporter Molecule

**Figure 3:**
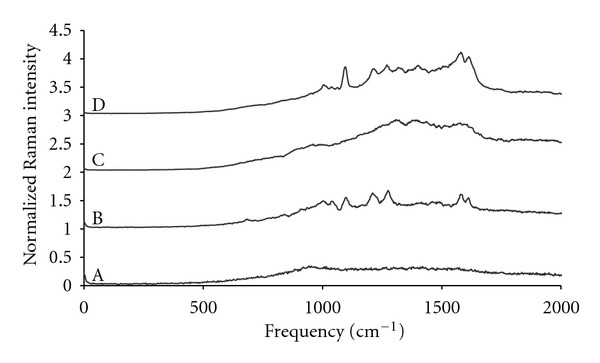
Normalized and offset Raman spectra during *in vitro* sensor development. (A) Raman spectrum of Au@Ag colloids. (B) Raman spectrum of 4-MPy adherent to Au@Ag colloids. (C) Raman spectrum of DNP adherent to Au@Ag colloids. (D) Raman spectrum of targeted nanosensor with DNP and 4-MPy ligands. *Y*  axis is arbitrary intensity units. Reprinted with permission from Nowak-Lovato et al. [11] (copyright 2009 Society for Applied Spectroscopy).

 To complement the previous studies regarding 4-MPy ligand interference with targeting, the next studies demonstrated minimal interference of DNP with the nanosensors ability to sense pH.  Figure 3 shows representative *in vitro* SERS spectra of targeted nanosensors at various stages of development.  Figure 3(A) shows a spectrum of unmodified Au@Ag nanoparticles which has no significant Raman signatures.  Figure 3(B) shows the spectrum of Au@Ag-4MPy which shows a variety of peaks including the peaks near 1600 cm^−1^ of primary interest and generally agrees with those reported elsewhere [7, 21].  Figure 3(C) represents the SERS spectrum of Au@Ag-DNP which demonstrates weak Raman peaks; however, these peaks are of a decreased intensity most likely due to the fact that DNP is conjugated to lysine, and further in distance from the nanoparticle surface. The distance of DNP from the particle could be contributing to the weakened DNP Raman signals [49].  Figure 3(D) represents a SERS spectrum of Au@Ag-4MPy-DNP which shows general agreement with Figure 3(B) with a mild contribution from DNP. 

**Figure 4:**
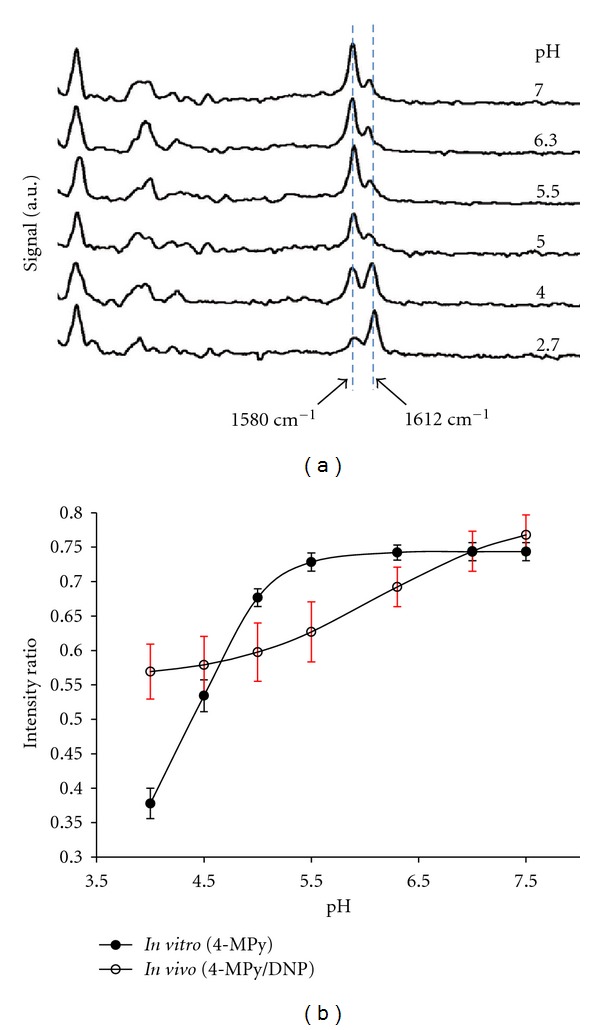
(a) Normalized and offset Raman spectra of Au@Ag-4MPy nanoparticles in varying pH-buffered solutions. (b) *In vitro *(closed circle) calibration curve of Au@Ag-4MPy nanosensors and* in vivo *(open circle) calibration curves of Au@Ag-4MPy-DNP nanosensors inside RBL-2H3 cells in pH-buffered media containing nigericin. *Y*-axis represents ratio of integration of peak centered at 1580 cm^−1^ versus the additive integration of both the peak centered around 1580 cm^−1^ and 1612 cm^−1^. Vertical error bars are dependent on approximately 100 pixels per image, repeated five times.

Many bands associated with 4-MPy seem to display a certain degree of pH sensitivity [7]. In particular, vibrational modes at frequencies of 1580 cm^−1^ and 1612 cm^−1^ display proportional ratiometric effects dependent upon pH. The vibrational modes of the pH-sensitive 4-MPy species are based on protonation and deprotonation of the ring N atom in the 4-MPy molecule [7]. Protonation of the molecule has been assigned to vibrational mode 1612 cm^−1^ while the unprotonated form has been assigned to 1580 cm^−1^. The peaks do not appear to spectrally shift with changing pH in this range. Calculating the ratio between the two frequency modes demonstrates a value that can be correlated to the pH of the molecule's environment. Since the two peaks to be rationed are spectrally close and completely dependent upon the same element (nitrogen) and whether it is bound or unbound to hydrogen, this sensor can accurately report pH without being tainted by the quantity of molecules present or by other molecular bond orientations. As is demonstrated in Figure 4(a), the intensity of the 1612 cm^−1^ peak is greatest at low pH and decreases while the pH is increased. The peak at 1580 cm^−1^ is greatest at high pH and decreases with decreasing pH. The spectra are representative of *in vitro* Au@Ag nanoparticles coated with 4-MPy on the surface. 

### 3.3. *In Vitro* and* In Vivo* Calibration

The *in vitro* calibration curve is shown in Figure 4(b) as closed circles. There is a potential that the pKa values of 4-MPy on the nanosensors will be different with and without the DNP added and also within cells. It is noted that previous work of measuring the point at which there is equal contributions of protonated and unprotonated species for *in vitro* nanosensors was 3.9 [50] and 4.0 [7]. It is known that the *in vitro* generation of calibration curves (signal versus concentrations) for biological measurements is of only limited use because of possible probe interactions with natural species inside live cells [40]. Measurements of dissociation constants,  *k*_*D*_, show that, for example, Ca^2+^ binding of dyes *in vivo* can be up to 5x than *in vitro* [51]. Fortunately, the generation of *in vivo* calibration curves is routine in fluorescence microscopy, and procedures for this application were similar. Briefly, multiple measurements are performed with varying levels of external concentrations of the metabolite of interest in the bulk media. The ionophore nigericin serves to equilibrate the external [H^+^] concentration with that of the cell [52]. To obtain *in vivo* calibration data, many measurements similar to those seen in Figure 4(a) are used to generate a calibration curve as shown in Figure 4(b) as the open circles. The error bars for the frequency ratios represent the standard deviation of the repeated measurements and generally are more precise at higher pH values. Note the dramatic shift in the apparent isosbestic point of the two curves. In addition, the ratio of the bands does not appear to flatten at zero with high acidity. This is likely arising from the background signal from DNP shown in Figure 3(D). However, all calibrations performed were with the addition of DNP to account for this effect. Vesicles formed along the endocytic pathway have been shown to display pH concentrations from 4.5 to 7.5. This range agrees quite well with our calibration curve showing a relevant pH range from 4.0 to 8.0 pH units. These data were fit to a sigmoidal curve with the values shown in (1). This equation was used to convert SERS spectra to pH maps in the subsequent experiments as follows:

(1) y=0.80−0.241+exp⁡((x−6.1)/0.69).

**Figure 5:**
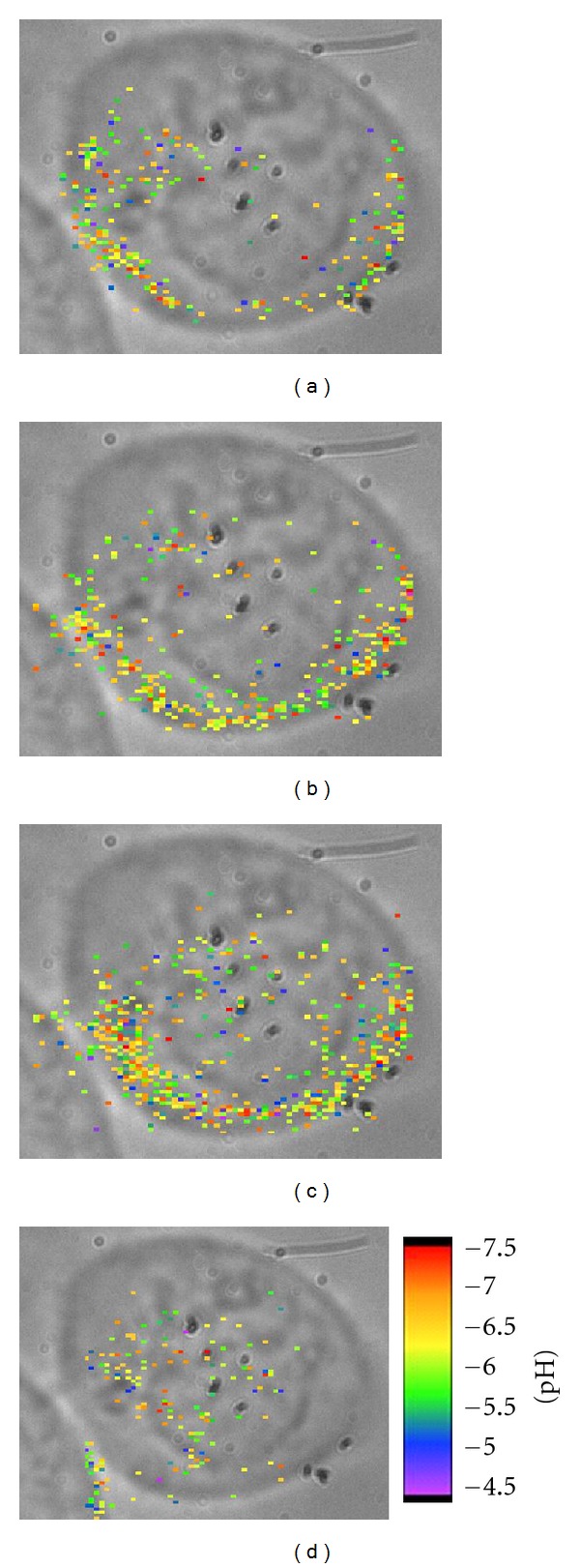
Time-lapse pH-calibrated images acquired every 3.5 min upon nanosensor addition up to 90 min. Calibrated pixels demonstrating pH are overlaid with bright field images. (a) 28 minutes, (b) 45.5 minutes, (c) 59.5 minutes and (d) 80.5 minutes. Reprinted with permission from Nowak-Lovato et al. [11] (copyright 2009 Society for Applied Spectroscopy).

Time-lapse, hyperspectral SERS imagery showing the evolution of targeted nanosensor delivery into the cells at 25°C is shown in Figure 5. One weakness of the microscope is that it only has the ability to acquire brightfield images at the beginning of a time-lapse image cycle. In addition, the brightfield image is a transmission, whole cell view at lower resolution than the pseudoconfocal, epi-acquired SERS image. Thus, the brightfield image only serves as a rough guide for cell structure and location prior to the initial Raman image.  Figure 5(a) shows the presence of targeted nanosensors twenty-eight minutes after the particles were added to the cells. The color-coded pixels represent the calculated pH values of the spectrum using the calibration curve shown in Figure 4(b). The colors correspond to the scale bar, with red and orange signifying neutral and near-neutral pH while blue and purple represent the most acidic locations. At the twenty-eight minute time point, most of the particles are near the edge of the cell membrane. There is a slight registration error between the brightfield image and the SERS image because the live cells, though adherent, are not immobile. Brownian motions on the order of 1-2 *μ*m can occur over the time course of a given experiment. However, it is noted that these nanoparticles have been or are about to be internalized because free-floating particles move too quickly to provide significant signal during the integrated acquisition time of the experiment.  Figure 5(b) shows the same scene at forty-five and a half minutes after addition. At this time point, there are significantly more particles internalized, although still the preponderance of the targeted nanosensors near the edge of the cell. In Figure 5(c), taken at fifty-nine and a half minutes, yet more particles have been delivered to the cells, and there is an increase of particles near the center of the cell. It is noted that the large nuclei of these cells could represent a “dead space” area where these targeted nanosensors, being inside endosomes, would not be found. The nucleus of the cell is just below center in the greyscale image but may be above the area of interrogation so that a more uniform distribution of particles is seen towards the surface of the cell. The SERS image at eighty and a half minutes, Figure 5(d), shows the majority of particles are away from the surface of the cell. These types of distributions of particles inside cells are also seen in the optical scattering experiments. 

**Figure 6:**
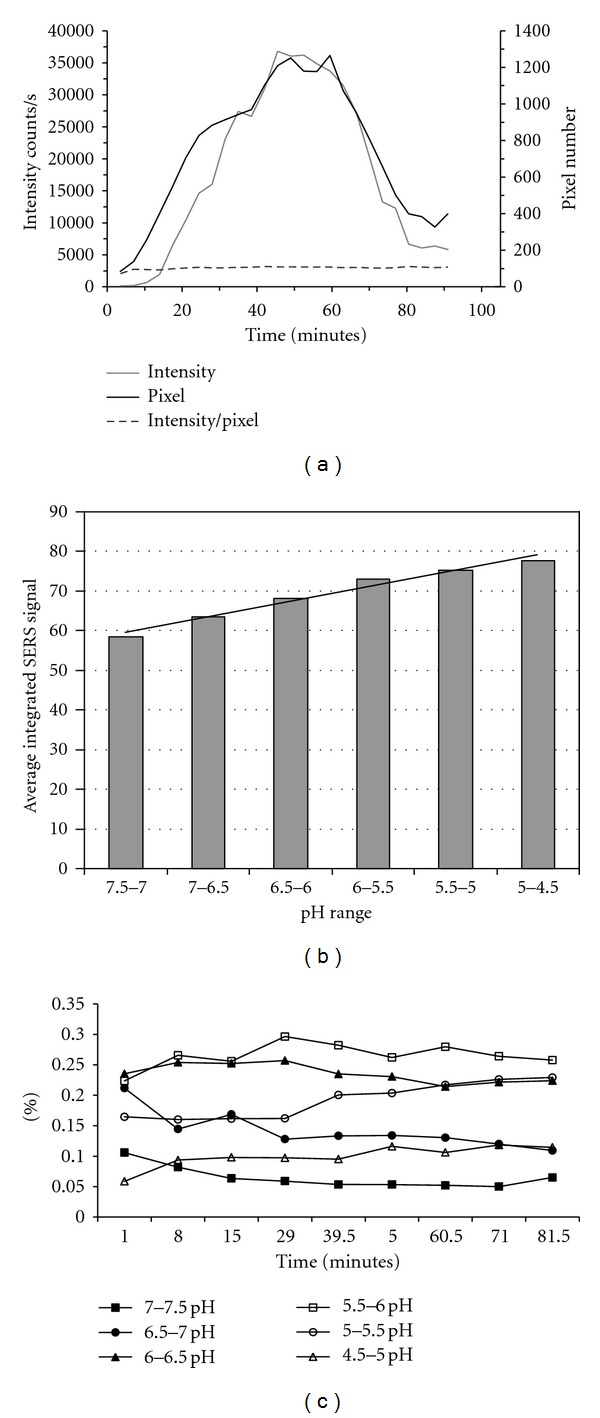
(a) Whole-cell image data ranging from 3.5 to 90 minutes. Total nanosensor intensity per image is depicted with gray line, corresponding to primary *y* axis. Total pixel count per image is shown with black line corresponding to secondary *y* axis. Average intensity per pixel is displayed with dashed line and also corresponds to secondary *y* axis. (b) Integrated SERS signal in the 1612 cm^−1^ range as a function of pH normalized to total pyridine signal and averaged overtime. Line is linear fit to the data, to guide the eye. (c) Percentage of pixels representing each pH grouping. (a and c) Reprinted with permission from Nowak-Lovato et al. [11] (copyright 2009 Society for Applied Spectroscopy), (b) Reprinted with permission from Nowak-Lovato and Rector [12] (copyright 2009 Thomson Corporation).

The number of pixels having significant SERS signal is plotted in Figure 6(a), representative on the secondary *Y* axis. There is most likely not a one-to-one correspondence between pixels measured and particles internalized. The measured size of each pixel is 0.5 *μ*m × 0.5 *μ*m which is also roughly the size of endosomes seen in this cell type [53]. The targeted nanosensors are below 0.2 *μ*m in size. Therefore, the targeted nanosensors encompass single pixels on average. However, it is uncertain if multiple nanosensors are present within an individual pixel. At fifteen minutes, approximately 400 pixels have SERS signal above the threshold. Over the course of an hour, the number of pixels with SERS signal swells to its peaks at approximately 1200. Over the subsequent thirty minutes, the number of pixels measured decreases to just fewer than 400. The rise in pixels over the first hour after nanoparticle delivery is explained by the slower cell processes expected to occur at 25°C compared to normal cellular growth temperature of 37°C. The nanosensors delivered suggest that the washing step did not completely remove free nanosensors. The drop in total pixels after one hour could possibly result from the degranulation process, where the nanosensors are excreted from the cells over time. In addition, it is possible that some of the nanosensors are being destroyed by the interrogation of the laser or being chemically modified by the harsh environment of the endosome. The possibility also exists that the nanosensors are aggregating, causing a decrease in pixel number without a loss of nanosensors. The total intensity of SERS signal per pixel and how this measurement corresponds to the numbers of nanosensors present was performed. The total signal intensity of the two pH sensitive 4-MPy peaks from 1500 cm^−1^ to 1626 cm^−1^ with a baseline subtraction from 900 cm^−1^ to 2200 cm^−1^ at each pixel was generated. All pixels with relevant SERS spectrum were used in the analysis. The primary *Y* axis is representative of the total SERS intensity counts for each image at the respective time point. The average intensity per pixel is also plotted in Figure 6(a) and aligns with the secondary *Y* axis just as the pixel number does. The trends for SERS intensity and pixel number appear to be quite similar. The average intensity per pixel plot depicts a nearly linear 1 : 1 ratio of SERS signal to pixel number. This data lends to elimination of nanosensors from the cells rather than aggregation. Since the ratio is not exactly 1 : 1, there might be a minor contribution of nanosensor aggregation or a slight difference in nanosensor size leading to minor signal enhancements. 

Comparison of integrated intensities of nanosensors over time and space indicated that most of the decrease in pixels seen is from elimination. Further, it would be ideal to be able to more specifically quantify the number of nanosenors within each endosome compartment so that endosome-specific information can be attributed, or that single endosome compartments can be followed over space and time. In Figure 6(b), the pH dependence of the integrated SERS 1612 cm^−1^ signal has been plotted and normalized to total pyridine signal and averaged over time. The pH dependence shows a near-linear trend that the integrated SERS signal increases with increasing acidity by approximately 23% when changed from 7.0–7.5 range to 4.5–5.0 range. The percentages of particles within pH categories which correspond to hypothetical states in the endosome maturation pathway are recorded over time [54]. These data are illustrated in Figure 6(c). The pH of the media used is approximately 7.4. It is assumed that, immediately after internalization, the endosome would have the same pH as the surrounding media. In the cell under interrogation, immediately there are about 10% of the pixels in the pH = 7.0–7.5 category and 23% of the pixels in the pH = 6.5–7.0 category. In both categories, the percentages of particles in these categories drop by 80 minutes to 6% and 11%, respectively. This trend can be attributed to internalized endosome maturing to lower pH values and recycled receptors having less particles available for new internalization processes. The particles representing the category of pH = 6.0–6.5, for the early endosome, begins the experiment at 23% of the population and remains constant throughout as particles entering and leaving this pH range are nearly balanced. The category of pH = 5.5–6.0, representing possibly the sorting endosome/early endosome, is the most populous category that begins in the twenties and increases to 30% over thirty minutes and remains increased throughout the experiment. The categories of pH = 5.0–5.5 and pH = 4.5–5.0, representing the late endosome/lysosomal stage, begins the experiment at 17% and 6%, respectively. Over time, both categories increase in relative abundance ending at 23% and 10%, respectively. This increase in abundance suggests that the bulk of the particles have been processed to the late endosome/lysosomal stage.

### 3.4. Temperature

**Figure 7:**
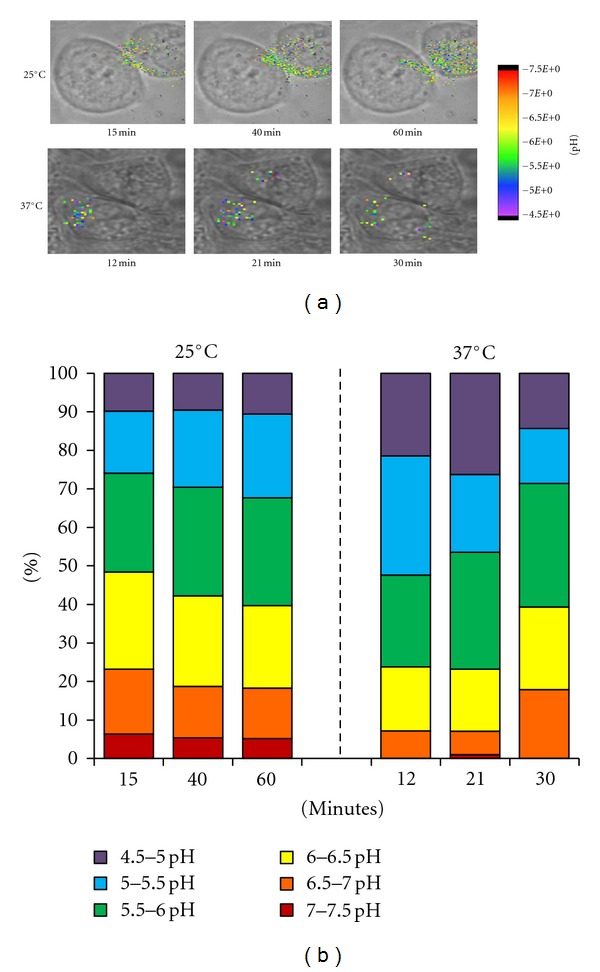
Temperature effects on nanosensors at 37°C versus 25°C. (a) Whole-cell pH image maps of cells incubated at 25°C with 10-minutes incubation of nanosensors, followed by imaging at 15′, 40′, and 60′ after the initial nanosensor addition. Bottom series are cells incubated at 37°C with 10-minute incubation with nanosensors followed by imaging at 12′, 21′, and 30′ after initial nanosensor addition. (b) Bar graphs demonstrating percentage of nanosensors from whole, in each pH grouping, at specified time points. Cells were incubated for ten minutes with nanosensors and then imaged at specified time points. Reprinted with permission from Nowak-Lovato et al. [11] (copyright 2010 Springer).

SERS-targeted nanosensors were added to cells held at either 37°C or 25°C.  Figure 7(a) depicts brightfield cellular images overlaid with color-calibrated SERS images and cells incubated at the two temperatures. The brightfield images are all recorded at initial time points before the first corresponding SERS image; minor alignment issues in the overlays correct for modest movements of the adherent cells. At the physiological temperature of 37°C, Fc*ε*RI crosslinking leads to marked endocytosis of the receptors over a 2–12-minute time period.

As expected, there was a considerable delay in receptor internalization in cells incubated at 25°C (Figure 7(a)). Accordingly, the imaging time was extended to over sixty minutes. The images in Figure 7(a) give a qualitative picture of the nanosensors and the pH of their localized environment.  Figure 7(b) averages data from three cells, with the goal of reporting the total nanosensor numbers in each pH grouping as a percentage of the whole.  Figure 7(b) also illustrates the expeditious movement of nanosensors through the endocytic pathway when incubated at 37°C. The nanosensor groupings devised from the images show that, at twelve minutes, 80% of the nanosensors are in pH groupings indicative of late endosomal/lysosomal vesicles. At later times, the overall number of sensors is reduced, consistent with trafficking of nanosensors to the cell surface via both the recycling compartments and by exocytic release of secretory lysosomes, followed by dissociation into the medium. A pool of nanosensors is detected with pH 6.0–7.0 compartments at these later times, suggestive of their retention within early sorting and recycling endosomes.

Experiments performed at 25°C demonstrate the temperature dependence of both initial receptor-mediated internalization of sensors and their slower progress through the endocytic pathway. When compared to cells incubated at 37°C, it is notable that nanosensors tend to accumulate in the pre-endosomal/recycling endosomes at the lower temperature. There is also a notable retention of the total number of nanosensors within the lowest pH compartments, as expected if the low temperature slows the arrival of receptor-nanosensor complexes into secretory lysosomes and their subsequent return to the plasma membrane by exocytosis. 

### 3.5. Pharmacologics

**Figure 8:**
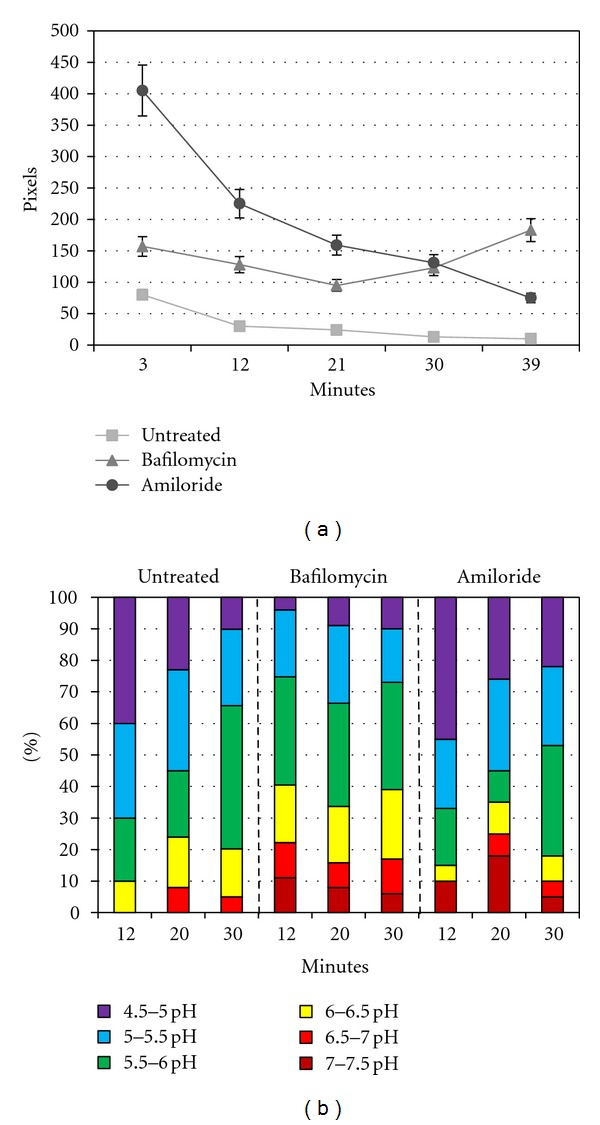
Pharmacologic effects on nanosensors at 37°C. Cells incubated at 37°C with the addition of bafilomycin or amiloride for two hours, followed by the addition of nanosensors. (a) Demonstration of total pixel/nanosensor number averaged from three individual cells. Cells are incubated at 37°C untreated or treated for two hours with drug, followed by the addition of nanosensors for ten minutes, then imaged. (b) Demonstration of the percentage of nanosensors averaged from three cells, from the whole, within the assigned pH groupings. Reprinted with permission from Nowak-Lovato et al. [11] (copyright 2010 Springer).

The targeted nanosensors were validated through a pharmacologic approach. First, the ability of the nanosensor to detect *increases* in pH within endosomes of cells treated with bafilomycin, an inhibitor of the H^+^ ATPase pump that pumps H^+^ into the endosomal vesicle [55–57], was tested. These results were compared with those in cells treated with amiloride, a known inhibitor of the Na^+^/H^+^ exchanger. This results in impairment of H^+^ exit from the endosomal vesicle and a *decrease *in endosomal pH [58–60]. In both cases, cells were incubated at 37°C with or without drugs for two hours prior to ten-minute exposure of cells to nanosensors and imaging in the continued presence of drugs. Results are reported in Figure 8. 

Figure 8(a) shows the total uptake of nanosensors under each condition, reported as the average number of Raman active pixels, at each time point, for at least three different cells. As stated previously, based on nanosensor size (<0.2 *μ*M) and pixel size (0.5 *μ*M × 0.5 *μ*M), it is assumed that there is at least one nanosensor per active pixel with a possibility for multiple nanosensors to be present per pixel (Figure 6(a)). Untreated cells accumulated approximately 100 nanosensors per cell during a 10-min exposure estimated based on assuming a 1 : 1 correlation of active pixel to the presence of at least one nanosensor. This value slowly decreased in number after washout, likely due to a combination of (1) recycling back to the surface, (2) directed trafficking to secretory lysosomes followed by egress of receptor-bound nanosensors through exocytosis, and (3) lysosomal degradation. Although the relative contributions of these two pathways are not distinguishable under these experimental conditions, it is possible to infer the locations of nanosensors from their distribution within compartments with specific pH at intervals during the chase (Figure 8(b)). At 12 minutes of chase, 70% of nanosensors were in compartments with pH of 4.5–5.0 (40%) or 5.0–5.5 (30%). Again, selective loss of this pool is consistent with arrival in the secretory lysosome, where they may be degraded or routed to the surface by exocytosis. 

Remarkably, amiloride-treated cells accumulated 4-fold more nanosensors (400/cell) within the same time of exposure (Figure 8(a)). It can be speculated that this large increase in number may be attributed to increased acidification of early and recycling endosomes, shifting the balance of receptors from the recycling pathway to the lysosome-directed pathway. Disruption of the Na^+^/H^+^ exchanger may also partially block the internalization process, leaving sensors bound to receptors at the cell surface where the medium is buffered at pH 7.2. This is illustrated in Figure 8(b), where 10–18% of sensors register a pH of 7.0–7.5 for up to 20 min of chase. Other notable observations in amiloride-treated cells include a detectable increase in nanosensors within very low pH compartments (pH 4.5–5) [58–60] and marked overall decline in number of nanosensors over the 40 min chase period. This is consistent with degradation with low pH compartments and/or exocytosis. 

Bafilomycin-treated cells took up an average of 150 nanosensors during the 10 min exposure, with no obvious decline over the 40-min chase period (Figure 8(a)). As shown in Figure 8(b), these nanosensors are trapped in compartments with significantly higher pH, consistent with blockade of the proton pump. Approximately 40% of the sensors reside in pH environments of 6.0–7.5 (red-orange-yellow bars in Figure 8(b) for the duration of the experiment). This may reflect occupancy in endosomal and recycling compartments with aberrant pH or retention of some receptors at the cell surface [61, 62]. Others have suggested that bafilomycin affects endosomal acidification primarily from the late endosomal to lysosomal stage [63–65]. Accordingly, less than 10% of sensors sense typical lysosomal pH in the range of pH 4.5–5.0. It seems likely that the sustained levels of nanosensors are due to either poor delivery to lysosomes and/or protection from degradation because of the pH dependency for lysosomal enzyme activity.

**Figure 9:**
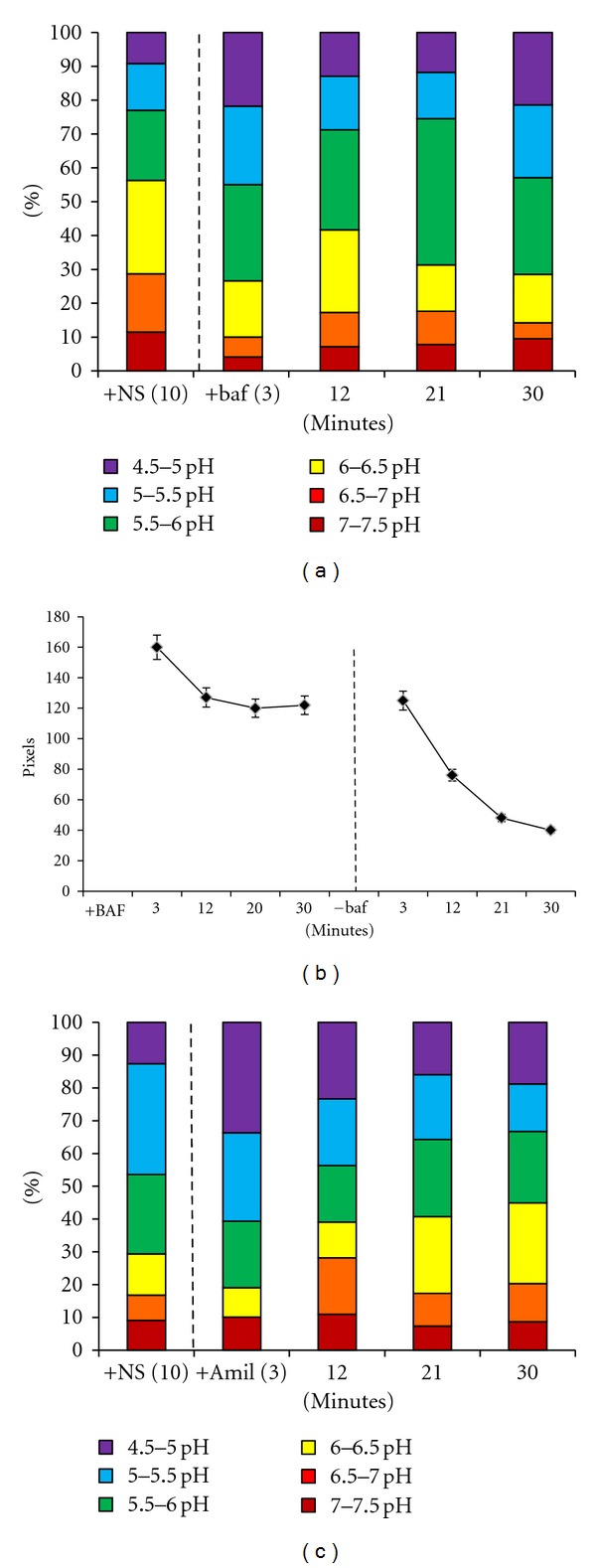
Nanosensor recovery from pharmacological treatment. (a) Representation of cells incubated at 37°C with nanosensor addition and incubation for ten minutes. The first bar of the graph is representative of nanosensors inside cells without chemical addition. After the dashed line, the bar graph is representative to nanosensor ph after the addition of bafilomycin to the cells. (b) Total pixel/nanosensor count within cells. Time points after dashed line are representative to the removal of bafilomycin and addition of fresh media. (c) Representation of nanosensor pH in cells incubated at 37°C with nanosensor incubation for ten minutes followed by the addition of amiloride. The first bar of the graph is cells incubated with nanosensors for ten minutes. The data after the dashed line is the addition of amiloride to the media followed by imaging at respective time points. Reprinted with permission from Nowak-Lovato et al. [11] (copyright 2010 Springer).

The protocol was varied in two ways: by (1) uptake of nanosensors by untreated cells at 37°C, followed by the addition of drugs and imaging or (2) treatment with drugs and nanosensors, followed by the removal of the drugs and imaging to follow recovery of compartments.  Figure 9(a) shows the distribution of nanosensors immediately after bafilomycin was added to the cells exposed to ten minute incubation with nanosensors. Graphs in this figure show that the effects of bafilomycin are apparent within minutes. This is most dramatic when comparisons are made at the 12-minute time point where only 25% of sensors are in lowest pH (4.5–5.5) environments compared to 70% in normal cells (see Figure 8(b)). Importantly, acute exposure to bafilomycin after uptake prevents loss of nanosensors. This is demonstrated in Figure 9(b), where the total number of nanosensors is stabilized within 12 minutes of bafilomycin treatment. This effect is reversible, as shown in Figure 9(b), where cells were treated for 30 min with bafilomycin followed by exchange with fresh media without drug. 

Acute effects of amiloride treatment on cells were also examined in Figure 9(c). Cells were again incubated with nanosensors for ten minutes at 37°C, followed by wash and addition of amiloride to the media (dashed line). Although 30% of nanosensors reach a low pH (4.5–5.0) environment by 3 min, there is a significant shift to higher pH environments within 12 minutes. This change appears to be complete within 20 minutes since the range of pH compartments is similar for both 20 min and 30 min of amiloride treatment. 

### 3.6. Pharmacologics and Temperature

**Figure 10:**
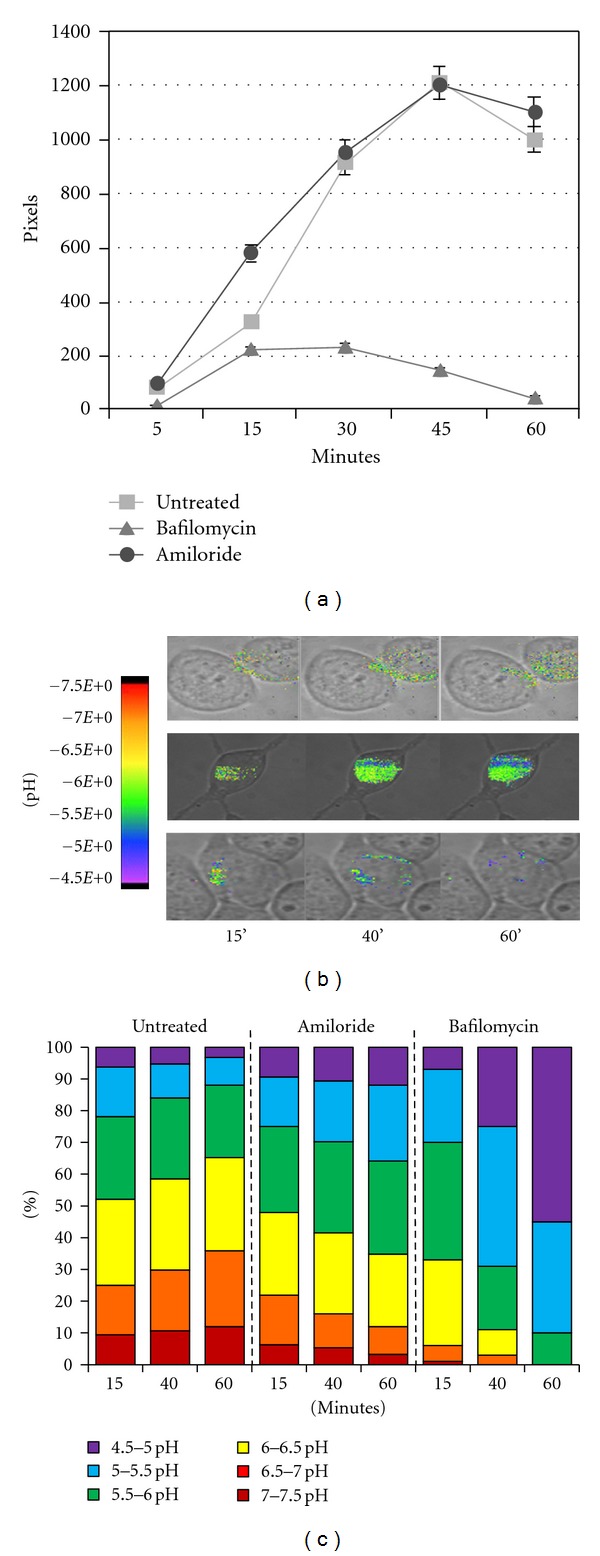
Pharmacologic effects on nanosensors at 25°C. Cells incubated at 25°C with the addition of bafilomycin or amiloride. Cells are incubated at 25°C with simultaneous addition of nanosensors and respective drug. (a) Is a demonstration of total nanosensor number displayed as pixels, averaged from three individual cells. (b) Whole-cell pH image maps of cells incubated at 25°C with the addition of nanosensors alone (untreated), with bafilomycin, or amiloride, followed by imaging at the corresponding time points below. (c) Bar graph demonstration of the percentage of nanosensors averaged from three cells, from the whole, that fall into each pH grouping in cells that are either untreated and incubated at 25°C, or treated with drug and incubated at 25°C. Reprinted with permission from Nowak-Lovato et al. [11] (copyright 2010 Springer).

The final set of experiments compared the uptake and transiting of nanosensors in cells pretreated with amiloride or bafilomycin but held at 25°C for all treatments. Under these conditions, untreated cells and amiloride-treated cells show a slow uptake of nanosensors, reaching similar plateau levels at approximately 45 min (Figure 10(a)). In contrast, bafilomycin-treated cells accumulate markedly fewer nanosensors per cell (Figure 10(a)) when held at room temperature. 

Analysis of the pH environment for internalized receptors under each condition suggests complex effects of low temperature on receptor trafficking through the endosome-lysosome systems. A spatial view of nanosensor localization within the untreated and treated cells at 15, 40, and 60 minutes is demonstrated (Figure 10(b)). In untreated cells, the slow uptake is accompanied by marked lack of delivery to low pH (4.5–5.5) compartments. Even after 60 minutes, about 10% of receptors would appear to have reached late endosomes or lysosomes (Figure 10(c)). Interpretation of receptor progression in bafilomycin-treated cells is complicated by the poor overall uptake. However, it is notable that over 35% of nanosensors taken up by amiloride-treated cells reside in a pH 4.5–5.5 environment within 60 min of uptake at 25°C. It cannot be distinguished whether these nanosensors have reached lysosomal compartments at this stage or whether they reside in endosomal compartments that have dramatically acidified after amiloride blockage of the Na^+^/H^+^ exchanger on their membranes. 

## 4. Conclusion

The development of targeted SERS pH nanosensors and the first applications of this technology have been demonstrated in live cells using time lapse Raman spectral microscopy. These experiments demonstrate that targeted nanosensors can be taken up by receptor-mediated internalization, providing a mechanism to simultaneously measure endocytic vesicle pH and follow the temporal and spatial progression of receptors as they traffic through the endosome-lysosome system. The ligands used for targeting and sensing have been shown to not significantly interfere with each other's function. As is demonstrated, confocal hyperspectral SERS whole-cell imagery can be rapidly acquired overtime. The imagery, with *in vivo* calibration data, can be used to create whole-cell, time-dependant localized pH maps. The targeted nanosensors show the markedly slow progression of receptors through the endocytic pathway when cells are held at 25°C. The targeted nanosensors reported the effects of H^+^ flux inhibiting drugs amiloride and bafilomycin on the endocytic compartments, providing reliable measurements of changes in lumenal pH. This technology represents a novel approach to use live cells as microlaboratories by developing nanosensors based on a nanoparticle platform and an abundance of ligand possibilities for sensing and targeting. The versatility of these probes shows promise for future applications relevant to intracellular trafficking and intelligent drug design.
